# Supplementary material for: Trends and risk factors associated with stillbirths: A case study of the Navrongo War Memorial Hospital in Northern Ghana
Source: PLoS One. 2020 Feb 21;15(2):e0229013. doi: 10.1371/journal.pone.0229013 (PMC7034822; doi:10.1371/journal.pone.0229013)
Supplement: Attachment — Submitted filename: Response to reviewers_25 Nov 2019.pdf.docx [file pone.0229013.s002.docx]

**Response to Editor’s Comments**

| **Editors comments** | **Response** |
| --- | --- |
| Please ensure that your manuscript meets PLOS ONE's style requirements, including those for file naming. The PLOS ONE style templates can be found at | We appreciate this important reminder by the editor and have now ensured that the entire manuscripts conforms to PLOS ONE style requirements |
| Please provide additional details regarding participant consent. If the need for consent was waived by the ethics committee, please include this information. If the data was accessed anonymously the lack of waiver will be acceptable. | Thanks to the editor for this point. We have now clarified this in the below statement: *“Because secondary data was used, informed consent was waived. Further to this, we ensured confidentiality and anonymity in data extraction,*  *processing/cleaning and analyses.”* |
| Please amend either the abstract on the online submission form (via Edit Submission) or the abstract in the manuscript so that they are identical. | We will do just this to ensure the abstract in the manuscript is identical to the online submission form |

**Response to Reviewers Comments**

| **Reviewer #1** | |
| --- | --- |
| **Comments** | **Response** |
| The manuscript is well written overall. Yet, the language quality should be verify, since punctuation marks issues can be identified over the manuscript, compromising the structure, organization of the text, leading to ambiguity, doubts and demanding extra attention to interpret the information. | We have edited the entire manuscript and to improve the language quality. We have also used  “grammar check” to improve the manuscript where necessary |
| The high quality methodology used in the present study contributes to the credibility of the results. Both statistical analysis and data collection are detailed and well described in the text, listing the different variables and criteria considered to elaborate the GEE regression models. However, I recommend a revision of some topics before the manuscript can proceed due to some minor issues. | We acknowledge and appreciate the reviewers kind comments |
| The introduction section brings great information about the topic and provides a contextualization of the subject to the reader. However, the given fact that “the rates of decline for South-East Asia and Africa are still very low” (page 5, 18th line) could be presented in a more reliable and solid manner if the “very low” numbers were introduced, according to the reference indicated. | We have now revised this statement to read as *“Saleem et al (2018) observed a 3.0% annual decline in stillbirth rate globally with much lower rates of decline for countries in South-East Asia and Africa. This the author observed will further result in the inability of these countries to achieve Every Newborn Action Plan goal of 12 per 1000births by 2030”* this can be found in page 5 lines 91-95 |
| At the beginning of the study setting section (page  7, 1st line), a retrospective study (Oduro et al, 2006) is used as a reference in order to describe the  Navrongo War Memorial Hospital (WMH)’s services - emergency obstetric care, surgical and other public health services. However, the referred paper does not describe it, stating only that “WMH is a district health facility that offers secondary clinical and public health services” (Oduro, 2006).  In addition, in the same paragraph (page 7, 10th line) the writer state that “The annual Antenatal attendance is about 2,500 and annual deliveries are | Thanks to the Reviewer for this observation. In our paper we have sought to expand or explain what Oduro et al 2006 meant by *“secondary clinical and public health services”.* These services include emergency obstetric care, surgical and other public health services as we observed.      We were of the view that since we have captured this in a previous paper, it suffices to cite this. We have however rephrased this as suggested by the |

| hovering around 1,500”. Meantime, the paper to which this sentence refers to (Nonterah et al, 2019) informs that this data was collected from an unpublished WMH material. Thus, it would be preferable if this same consideration was made in the present study, since this material is not available to the external scientific community. | Reviewer. *“Unpublished data from the hospital shows an average yearly Out Patient Department (OPD) attendance of about 55,000 with an average of 10,000 in-patient admissions of which maternal related conditions form part of the top ten causes of admissions. The annual Antenatal attendance is about 2,500 and annual deliveries are hovering around 1,500”* |
| --- | --- |
| The applied definition of stillbirth as “any foetal loss after 28 weeks of gestation, and or a weight of  1000g (1kg)” (data analysis section – page 8, 1st line) is referring to a Lancet Global Health’s article (Blencowe et al, 2016). However, this is not the primary source of the stillbirth definition, but yes the International Classification of Diseases 10th revision (ICD-10). Also, according to the ICD-10, there is a third criterium to classify stillbirth: fetus of 35cm of length or more. If the writer decides not to include it, it is possible to justify the reasons for it (eg. absence of length data). | We appreciate the observation made by the Reviewer. We do agree that the primary source is ICD-10 and we have included this as a reference now. We have also justified the reason for nonexclusion of the third component of the definition in the following sentence: *“The ICD-10 further states the foetus must be 35cm of length or more [23]. We however had no information on the foetal length at birth”.*  This can be found in page 9 lines 173-174. |
| The table 1 is well presented but contains some minor issues. The variable “perineum” may not be self-explicative, demanding a more descriptive nomenclature, such as “perineum status”.  Still in the table 1, the variable “maternal parity” presents 3 categories: P0, P1-P5 and >P5. Despite the explanation about the variable in the method section, the categories could be presented in a clearer manner if the 3 nominal classifications were included: primiparous, multiparous and grand multiparous.  Also, the variable “gestational age” is presented with 3 categories: <37 weeks, 37 to 40 weeks and >40 weeks. However, the inclusion of the classification pre term and term (as previous presented in the text), with the corresponding intervals, may contribute to data understanding. | Perineum corrected to *“State of perineum”*      We have now amended this using the nominal classifications proposed by the Reviewer.  *“Primiparous” “multiparous” and “Grand*  *multiparous”*    We have similarly amended gestational age as *Preterm (<37 weeks), Term (38-40 weeks) and Post-term (>40 weeks)* |
| The results from the study are described in a very fine way. However, there are some important considerations in order to contribute to the quality | We indeed appreciate this observation and have made these amendments: The phrase “The proportion of teenage pregnancies (≤19 years) was |

| of this section. The phrase “The proportion of teenage pregnancies (≤19 years) was relatively high at about 15.1%” (6th line, page 12) may be reformulated to follow the results section requirements. The use of expressions such as “high” is considered an interpretation, what is an attribute of the discussion section. In addition, to discuss if it is a high or low rate, it is important to compare the values with national/international references. | relatively high at about 15.1%” has been edited to read *“About 15.1% of the participants were less than 20 years of age denoting teenage pregnancy”* |
| --- | --- |
| The discussion section highlights important previous findings, intertwining the existing literature and the risk factors associated with stillbirths from the presented study. However, in the discussion stablished about the C-section rate over the study period may be reconsidered in some aspects (page 18, 31st line). First, according to the text, the C-section rate of 12.5% is within the rate interval proposed by WHO (10 to 15%). Therefore, it may be considered as a positive result, providing discussions about the conditions of the Spontaneous Vaginal Delivery instead, which also requires skilled health personnel, good sanitary conditions and a satisfactory infrastructure environment. Second, the writers compare the Csection rate to the developed “world”, considering this last one as a “positive” parameter. However, developed countries usually present high C-section rates not only because of emergency obstetric care, but also due to many other factors that lead to unnecessary recommendations of this surgical procedure (eg. private health insurance interests; convenience of choosing the day of delivery). Therefore, when discussing about this specific result, it is important to conduct a careful discussion, considering all long-term benefits of the vaginal delivery. | We do appreciate the further explanation given by the Reviewer. We do not want to overemphasize the discussion on CS rates since the focus of the paper is stillbirth. We do agree with the Reviewer that there are several other reasons for high CS rates especially in high income countries. |
| The conclusions were drawn appropriately based on the presented data, which are fully available in the manuscript over the text, but also in tables and graphics. However, the affirmation “First time pregnant mothers and those carrying male foetus should strictly follow antenatal and prenatal | Thanks for this important point. We have now edited the conclusion and it reads:  *“The study showed modest decline in stillbirth rate over the study period. It is evident from the study that stillbirth rate is still relatively high and some* |

| services and recommendations as they are at a higher risk of stillbirths” may be reconsidered. Despite the study shows great findings, it is a retrospective non controlled study, carrying some important limitations. Even though the literature supports many of the findings, it is essential to analyze carefully all results. For example, the correlation between pregnancies of boys and stillbirth can suggest a fact but it is not correct to stablish strong recommendations about it without a deep interpretation. The results from scientific studies may imply early actions and miss interpretations, generating negative consequences. Therefore, I suggest that the conclusion section be revised and based on solid evidenced and scientific findings. | *neonatal and maternal characteristics are major contributors. Efforts aimed at impacting on stillbirths should include the elimination of outmoded cultural practices such as FGM. Health systems strengthening especially in the utilization of antenatal care services and skilled attendants at delivery are essential in improving foeto-maternal outcomes”.* |
| --- | --- |
| **Reviewer #2** | |
| I will suggest updating reference 1. | We believe the information contained in the reference is still relevant but we have added a current reference to it. |
| In line 13 of the background, I suggest abbreviating the word “Sub-Saharan Africa” in the first moment that it was presented. | We have corrected this now |
| Also, through the paper, the authors used many abbreviations that they did not use after, I suggest removing and only keeping the abbreviations that have more the one citation. | We have screened through and removed the abbreviations such as KNM, OPD, VA which were not repeated in the manuscript |
| I think it was not clear if the author used a crosssectional or longitudinal data. I understood that it was a cross-section study using trends. | Data were collected in serial cross-sectional manner |
| I think would be important to describe more about the “systematically documented information”, on the second and third line of the data collection section. | We have now expanded on this to read as:  “*The hospital keeps record books that contain systematically documented information on each patient attending the hospital. These hospital record books includes a maternal register that is used to capture information on all maternal related cases, information captured includes maternal demographic characteristics, mode of delivery, delivery outcome (live, fresh and macerated* |

|  | *stillbirths and birth weight), gestation at delivery, genital mutilation status of the mother*” |
| --- | --- |
| Also, I would like to know more information about the “A structured designed data capturing tool”.  Did the authors use a tool? Which tool was that? | This was a designed excel spread sheet |
| The author did not specify which period the data was collected, this information should be clear. | We have now clarified that “data were collected between 2003 and 2013” |
| The data was double coded but what is the reliability of the data? Could the author present a statistic measure for that? | We merged data and there was a complete match hence data were reliable |
| In the analyzes section, I notice that in the GEE analyzes the authors used sometimes the groups of high risk as a reference, for example, mothers age “12-19 yrs”, mother occupation “Unemployed”, gestation >37 weeks and sometimes groups with fewer risks as normal birth weight, my suggestion is using the groups with less risk as reference “1” and establish a pattern in the analyzes. | We have re-analysed this. For age *“20-34 years”* is now the reference group while for gestational age *“38-40 weeks (term)”* is now the reference group |
| Also, the author described what GEE means but they did not describe the assumption of the GEE. | We have now modified the below statement to capture the relevant assumptions for GEE that relates to our analyses.  *“The GEE formulated by Liang and Zeger (1986) was found to be the best regression approach for estimating the relationship of factors associated with stillbirth because of the correlation in our data which is an important assumption for using GEE [25]”* |
| I would like also the authors to discuss more the result's implications and recommendations. | We have expanded the conclusion to include recommendations:  *“The study showed modest decline in stillbirth rate over the study period. It is evidence from the study that stillbirth rate is still relatively high and some neonatal and maternal characteristics are major contributors. Efforts aimed at impacting on stillbirths should include the elimination of outmoded cultural practices such as FGM. Health systems strengthening especially in the utilization of antenatal care services and skilled attendants at delivery are essential in improving foeto-maternal* |
|  | *outcomes”.* |
| In the last paragraph of the discussion they mentioned that the data is longitudinal, I am confused about that. Do you follow the patients between 2003 and 2013? If you did follow, this information should be clearer in the method section. | We have now clarified the cross-sectional nature of our study |
| Please review the references for typos. | Thanks for the comment. We have now revised the relevant references thoroughly |
